# Elementa Physiologiæ Specialis Corporis Humani

**Published:** 1846-01

**Authors:** 


ART. XII.—*Elementa Physiologiæ specialis Corporis Humani.* Auctore AUGUSTO ARNOLDO SEBASTIAN, Ordinis Leonis Neerlandici Equite, &c. Editio altera. *Groningæ,* 1842. pp. 356.

THIS work is dedicated to his class by Professor Sebastian. The plan he has adopted is on the model of the older writers. The work is divided into numbered aphorisms, each concisely stating a fact, or a principle. There is nothing original in the work. It is a good compendium, and a work suitable to students.

